# A Primer on Learning in Bayesian Networks for Computational Biology

**DOI:** 10.1371/journal.pcbi.0030129

**Published:** 2007-08-31

**Authors:** Chris J Needham, James R Bradford, Andrew J Bulpitt, David R Westhead

**Affiliations:** Whitehead Institute, United States of America

## Introduction

Bayesian networks (BNs) provide a neat and compact representation for expressing joint probability distributions (JPDs) and for inference. They are becoming increasingly important in the biological sciences for the tasks of inferring cellular networks [1], modelling protein signalling pathways [2], systems biology, data integration [3], classification [4], and genetic data analysis [5]. The representation and use of probability theory makes BNs suitable for combining domain knowledge and data, expressing causal relationships, avoiding overfitting a model to training data, and learning from incomplete datasets. The probabilistic formalism provides a natural treatment for the stochastic nature of biological systems and measurements. This primer aims to introduce BNs to the computational biologist, focusing on the concepts behind methods for learning the parameters and structure of models, at a time when they are becoming the machine learning method of choice.

There are many applications in biology where we wish to classify data; for example, gene function prediction. To solve such problems, a set of rules are required that can be used for prediction, but often such knowledge is unavailable, or in practice there turn out to be many exceptions to the rules or so many rules that this approach produces poor results.

Machine learning approaches often produce better results, where a large number of examples (the training set) is used to adapt the parameters of a model that can then be used for performing predictions or classifications on data. There are many different types of models that may be required and many different approaches to training the models, each with its pros and cons. An excellent overview of the topic can be found in [6] and [7]. Neural networks, for example, are often able to learn a model from training data, but it is often difficult to extract information about the model, which with other methods can provide valuable insights into the data or problem being solved. A common problem in machine learning is overfitting, where the learned model is too complex and generalises poorly to unseen data. Increasing the size of the training dataset may reduce this; however, this assumes more training data is readily available, which is often not the case. In addition, often it is important to determine the uncertainty in the learned model parameters or even in the choice of model. This primer focuses on the use of BNs, which offer a solution to these issues. The use of Bayesian probability theory provides mechanisms for describing uncertainty and for adapting the number of parameters to the size of the data. Using a graphical representation provides a simple way to visualise the structure of a model. Inspection of models can provide valuable insights into the properties of the data and allow new models to be produced.

## Bayesian Networks

**Figure 1:**
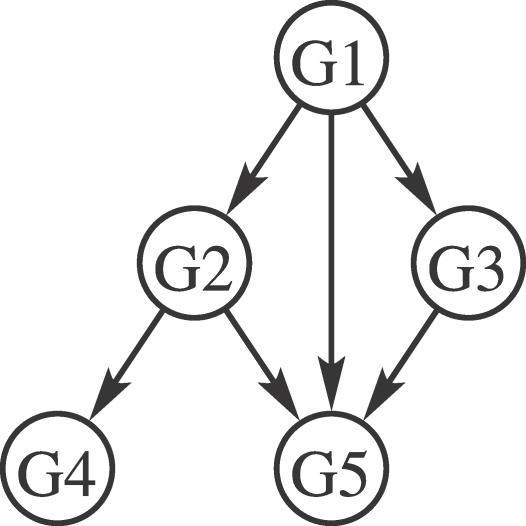
An Example: Gene Regulatory Networks Gene regulatory networks provide a natural example for BN application. Genes correspond to nodes in the network, and regulatory relationships between genes are shown by directed edges. In the simple example above, gene G1 regulates G2, G3, and G5, gene G2 regulates G4 and G5, and gene G3 regulates G5. The probability distribution for the expression levels of each gene is modelled by the BN parameters. Simplification results from the fact that the probability distribution for a gene depends only on its regulators (parents) in the network. For instance, the expression levels of G4 and G5 are related only because they share a common regulator G2. In mathematical terms, they are conditionally independent given G2. Such relationships lead to factorisation of the full JPD into component conditional distributions, where each variable depends only on its parents in the network. *p*(G1, G2, G3, G4, G5) = *p*(G1)*p*(G2|G1)*p*(G3|G1)*p*(G4|G2)*p*(G5|G1, G2, G3)

In a graphical model representation, variables are represented by nodes that are connected together by edges representing relationships between variables. Figure 1 provides an example of a BN describing a gene regulation network. The expression of each gene is represented by one variable of a JPD that describes how the genes are regulated by each other. Such a JPD may be complex even for just five variables; however, the graphical representation makes it clear where the regulatory relationships exist between the genes.

For BNs, the edges of the graph must form a directed acyclic graph (DAG)—a graph with no cyclic paths (no loops). This allows for efficient inference and learning. JPDs can be expressed in a compact way, reducing model size through exploiting conditional independence relationships—two variables are conditionally independent if they are independent given the state of a third variable. A benefit of BNs is that they may be interpreted as a causal model which generated the data. Thus, arrows (directed edges) in the DAG can represent causal relations/dependencies between variables. However, it must be noted that to learn a causal model from data needs more than association data, and this is discussed toward the end of this primer under the heading Causality.

Bioinformatics applications of BNs have included gene clustering and the inference of cellular networks [1], since they are well-suited to modelling stochastic complex biological systems, and the resulting networks can be easily understood. An excellent example of combining data and domain knowledge in the bioinformatics field is the MAGIC BN which has been designed using expert knowledge for combining information from diverse heterogeneous data sources for the classification task of gene function prediction [3].

### 

#### Conditional probability distributions (model parameters).

The relationships between variables are encoded by conditional probability distributions (CPDs) of the form *p*(*B|A*)—the probability of *B* given *A*. For *discrete variables,* probability distributions are expressed as conditional probability tables (CPTs) containing probabilities that are the model parameters (see Figure 7 and related text for examples). For each node, the probability that the variable will be in each possible state given its parents' states can be calculated based on the frequency observed in a set of training data. It is often useful/necessary to use a prior distribution for the model parameters, as, without a prior, a possible configuration that was not seen in the training examples would be incorrectly assigned a zero probability of ever being observed. (Equally well, these probabilities may be estimated by an expert and used alongside those learned from data).

**Figure 2:**
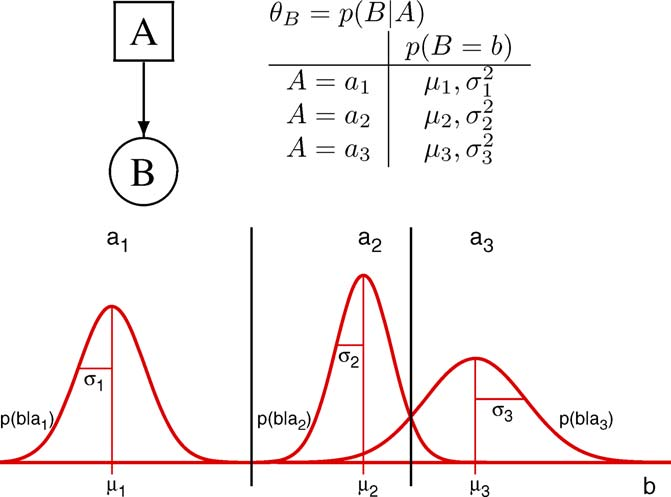
Illustration of Model Parameters for Two-Node Bayesian Network

For BNs, which use *continuous variables,* conditional probability densities are used in a similar way to CPTs. Figure 2 presents a simple BN which introduces the concept of using continuous variables. The usual notation is to use squares for discrete nodes and circles for continuous nodes. A continuous node, *B*, with a discrete parent, *A*, (say, a variable with *k =* 3 states) leads to a model of the continuous data using *k* Gaussian distributions. Thus, given that *A* is in state *a_i_*, the likelihood of a value of B may be inferred, or, alternatively, given a value *b* for variable *B*, the probability that variable *A* is in state *a_i_* may be inferred. Parameters for the Gaussians (or other distributions) can be learned from training data. θ*_B_* is the parameter set that encodes the model for *B* in terms of three Gaussians, one for each of the three possible states of *A*. A mean *μ_i_* and standard deviation *σ_i_* are the parameters for the Gaussian distribution which models *p*(*b|a_i_*).

In a similar way, regression models for CPDs of continuous variables with continuous parents may be used. In this case, θ*_B_* = *P*(*B|A*) ∼ *N*(*c + ma, σ* ^2^). i.e., the CPD for *B* is a Gaussian distribution with a mean dependent on the value of *A = a*, with constants *m* and *c* determined by regression of *B* on *A*.

#### Joint probability distributions.

It is the JPD over all the variables that is of great interest. However, the number of model parameters needed to define the JPD grows rapidly with the number of variables. Through exploiting conditional independence between variables, the models may be represented in a compact manner, with orders of magnitude fewer parameters.

Relationships between variables are captured in a BN structure *S* defined by a DAG (as in the gene regulatory network example in Figure 1). This enables the JPD to be expressed in terms of a product of CPDs, describing each variable in terms of its parents, i.e., those variables it depends upon. Thus:

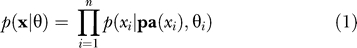
where **x** = { *x*_1_, … , *x_n_* } are the variables (nodes in the BN), and θ = { θ_1_ , … , θ*_n_* } denotes the model parameters, where θ*_i_* is the set of parameters describing the distribution for the *i*th variable *x_i_*, and **pa**(*x_i_*) denotes the parents of *x_i_*. Each parameter set θ*_i_* may take a number of forms—commonly a CPT is used for discrete variables, and CPDs (such as Gaussian distributions) are used for continuous variables. Classification/regression models can be used to learn the parameters for each node in the network.

#### Inference in Bayesian networks.

For the known BN structure (gene regulatory network) in Figure 1 and a CPD for each node (modelling gene interactions), given evidence about the expression levels of some genes, inferences about the likely values of other genes can be made. For example, the value of G1 may be inferred from the values of the other genes, i.e., *p*(G1|G2, G3, G4, G5). More generally, inferences of the values of a set of variables may be made given evidence of another set of variables, by marginalising over unknown variables. (Marginalising means considering all possible values the unknown variables may take, and averaging over them.) Simple inference examples are illustrated in the next section.

Conceptually, inference is straightforward, *p*(*x|y*) is calculated as a product of relevant CPDs, using Bayes rule [*p*(*a|b*) *= p*(*b|a*)*p*(*a*)*/p*(*b*)] to calculate any posterior probabilities. Computationally, the calculation of inference in this way is hard and inefficient. A number of methods exist that exploit the structure of the graph to derive efficient exact inference algorithms such as the sum–product and max–sum algorithms. For many problems, however, exact inference is not feasible, and, therefore, the use of approximation methods such as variational methods and sampling approaches are required.

#### Conditional independence.

Two variables are conditionally independent if they are independent given the state of a third variable. Mathematically, *a* and *b* are conditionally independent given *c* if:

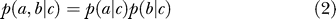



Conditional independence relationships are encoded in the structure of the network, as illustrated in the three cases below. Regulation of three genes *x*, *y*, and *z* is taken as an example. In each case, the situation is described, along with a BN diagram, an equation for the JPD, and an equation for inference of *p*(*z|x*).

**Figure 3:**
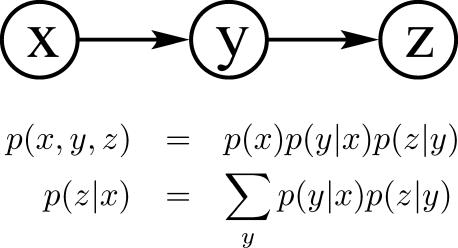
Serial Connection

*Serial connection.* For example, when gene *x* promotes gene *y*, and gene *y* promotes gene *z* (Figure 3). In this case, evidence is transmitted unless the state of the variable in the connection is known: if the expression level of gene *y* is unknown, then evidence of the level of *x* effects the expected level of *z*; if *y* is known, then the level of *z* depends only on the expression level of *y*. *z* is conditionally independent from *x*.

**Figure 4:**
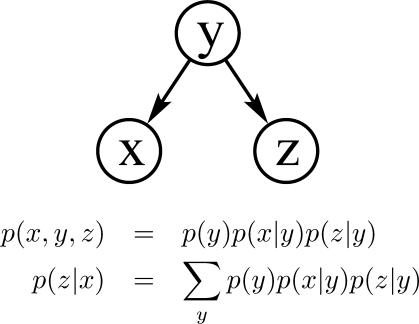
Diverging Connection

Diverging connection. For example, when a transcription factor *y* turns on two genes *x* and *z* (Figure 4). As with a serial connection, evidence is transmitted unless the variable in the connection is instantiated: if the expression level of *y* is unknown, then evidence of the level of *x* effects the level of *z* (since they are co-regulated—if *x* is highly expressed, then the likely level of *y* may be inferred, which in turn would influence the expression level of *z*); if *y* is known, then the level of *z* depends only on the expression level of *y*. *z* is conditionally independent from *x*.

**Figure 5:**
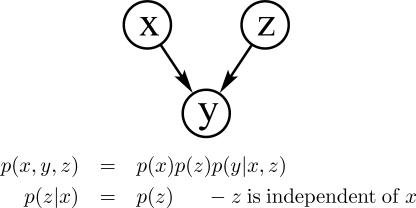
Converging Connection

Converging connection. For example, when two genes *x* and *z* both promote gene *y* (Figure 5). Evidence is transmitted only if the variable in the connection or one of its children receives evidence: if *y* is unknown, then evidence of the expression level of gene *x* does not help to infer the expression level of *z*—*x* and *z* are independent; however, if *y* is known, then the level of *x* does help to infer the expression level of *z*. Importantly, at the v-structure in the network, the CPD for *y* encodes the dependency of *y* on both *x* and *z*. Note in this case that *p*(*x,z|y*) *≠ p*(*x|y*)*p*(*z|y*).

In the case of a converging connection, it is also worthwhile noting that when the value of *y* is known as well as *x*, then this evidence helps to infer the value of *z*, and *x* and *z* are no longer independent variables:

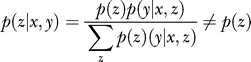



Thus, the structure of the model captures/encodes the dependencies between the variables and leads to a different *causal* model.

#### An example: Naïve Bayes classifier for interaction site prediction.

As a simple example, consider the task of predicting interaction sites on protein surfaces from measures of conservation and hydrophobicity of surface patches. This gives three variables: *I,* whether the patch is an interaction site or not; *C,* conservation score for the patch; and *H,* the hydrophobicity of the patch. *I* is a discrete class variable. Both *C* and *H* are continuous variables (though may be quantised to form discrete data). Conservation and hydrophobicity are both reasonably good predictors of interaction sites, and the information from these independent predictions may be combined in a naïve Bayes classifier to improve performance. The structure of the model for a naïve Bayes classifier has a class node (the one to be inferred from the other observed variables) as a parent to all other independent variables and is illustrated in Figure 7. Such a model structure is excellent for integrating information, and for maintaining a small model size. [For a set of *n* binary variables, a completely connected DAG has 2*^n^* − 1 free parameters, an inverted naïve Bayes classifier (where the class node depends on all other variables) has 2*^n^* ^−1^ + n + 1 free parameters, whereas a naïve Bayes classifier has only 2*n* + 1 free parameters! For a model with 100 binary variables, this is more than 2^90^ times smaller!]. In the next section of this primer, the learning of parameters for this simple example is illustrated. This example is inspired by [4] in which a naïve Bayes classifier is used within a classification scheme to predict protein–protein interaction sites using a number of predictive variables.

## Parameter Learning

The simplest approach to learn the parameters of a network is to find the parameter set that maximises the likelihood that the observed data came from the model.

### 

#### Likelihood.

In essence, a BN is used to model a probability distribution **X**. A set of model parameters θ may be learned from the data in such a way that maximises the likelihood that the data came from **X**. Given a set of observed training data, *D* = { **x**_1_, … , **x** *_N_* } consisting of *N* examples, it is useful to consider the likelihood of a model, *L*(θ), as the likelihood of seeing the data, given a model:

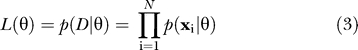
It should be noted here that **x**_i_ is the *i*th training example and that the likelihood of *D* being generated from model θ is the product of the probabilities of each example, given the model.

#### Maximum likelihood.

The learning paradigm which aims to maximise *L*(θ) is called *maximum likelihood* (ML). This approximates the probability of a new example **x** given the training data *D* as *p*(**x** *|D*) ≈ *p*(**x**|θ_ML_) where θ_ML_ is the maximum (log) likelihood model which aims to maximise ln *p*(*D*|θ), i.e., θ_ML_ = arg max*_θ_* ln *p*(*D*|θ). This amounts to maximising the likelihood of the “data given model.” ML does not assume any prior. Using negative log likelihood is equivalent to minimising an error function.

#### Maximum posterior.

In order to consider a prior distribution, a *maximum a posteriori* (MAP) model can be used. This approximates the probability of a new example **x** given the training data *D* as *p(* **x** *|D)* ≈ *p*(**x**|θ_MAP_) where θ_MAP_ is the MAP probability (likelihood of the “model given data”) which aims to maximise ln *p*(θ|*D*), i.e., θ_MAP_ = arg max*_θ_* ln *p*(θ|*D*)*.* This takes into account the prior, since through Bayes' theorem: *p*(θ|*D*) *= p*(*D*|θ)*p*(θ)*/p*(*D*).

Often ML and MAP estimates are good enough for the application in hand, and produce good predictive models. The numerical example at the end of this section illustrates the effects of ML and MAP estimates with different strength priors and training set sizes. Both ML and MAP produce a point estimate for θ. Point estimates are a single snapshot of parameters (though confidence intervals on their values can be calculated).

#### Marginal likelihood.

**Figure 6:**
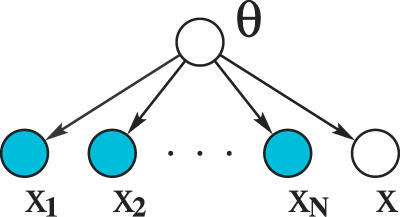
Graphical Model Illustrating Bayesian Inference

For a full Bayesian model, the uncertainty in the values of the parameters is modelled as a probability distribution over the parameters. The parameters are considered to be latent variables, and the key idea is to marginalise over these unknown parameters, rather than to make point estimates. This is known as marginal likelihood. The computation of a full posterior distribution, or model averaging, avoids severe overfitting and allows direct model comparison. In [8], Eddy introduces Bayesian statistics with a simple example, and integrates over all possible parameter values, illustrating a more rigorous approach to handling uncertainty. Formulating Bayesian learning as an inference problem, the training examples in *D* can be considered as *N* independent observations of the distribution **X**. Figure 6 shows a graphical model where the shaded nodes **x_i_** represent the observed independent training data and **x** the incomplete example observation for which the missing values are to be inferred, all of which are dependent upon the model θ.

The joint probability of the training data, the model, and a new observation **x** is:

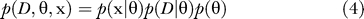
where *p*(θ) is the prior. Applying the sum rule [*p*(*a*) *= ∫p*(*a,b*)*db*]:

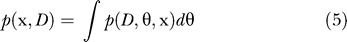



Applying the product rule [*p*(*a,b*) *= p*(*a|b*)*p*(*b*)] to the left-hand side, and substituting (4) for the joint probability on the right-hand side, then dividing both sides by *p*(*D*), gives the predictive distribution for **x**:

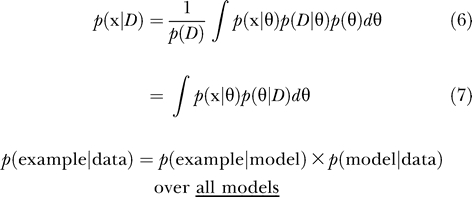



This is computing a full Bayesian posterior. In order to do this, a prior distribution, p(θ), for the model parameters needs to be specified. There are many types of priors that may be used, and there is much debate about the choice of prior [9]. Often the calculation of the full posterior is intractable, and approximate methods must be used, such as point estimates or sampling techniques. Marginal likelihood fully takes into account uncertainty by averaging over all possible values.

#### Learning from incomplete data.

The parameters for BNs may be learned even when the training dataset is incomplete, i.e., the values of some variables in some cases are unknown. Commonly, the Expectation–Maximisation (EM) algorithm is used, which estimates the missing values by computing the expected values and updating parameters using these expected values as if they were observed values.

EM is used to find local maxima for MAP or ML configurations. EM begins with a particular parameter configuration 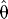 (possibly random) and iteratively applies the expectation and maximisation steps, until convergence.

*E-step.* The expected values of the missing data are inferred to form *D_C_*—the most likely complete dataset given the current model parameter configuration.

*M-step.* The configuration of 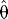 which maximises *p*(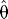 *|D_C_*) is found (for MAP).

Using EM to find a point estimate for the model parameters can be efficient to calculate and gives good results when learning from incomplete data or for network structures with hidden nodes (those for which there is no observed data).With large sample sizes, the effect of the prior p(θ) becomes small, and ML is often used instead of MAP in order to simplify the calculation. More sophisticated (and computationally expensive) sampling methods such as those mentioned below may also be applied to incomplete data. One advantage of these methods is that they avoid one of the possible drawbacks of EM—becoming trapped in local optima.

There may be cases of hidden nodes in gene regulatory networks, where the network is known, but experiments have not provided expression levels for all genes in the network—model parameters can still be learned. The ability to handle incomplete data is an important one, particularly when considering that expression data may come from different laboratories, each looking at different parts of a gene regulatory network, with overlap of some genes whilst others are missing. In this case, all the collected data can be used.

#### Sampling methods.

A number of sampling methods have been used to estimate the (full) posterior distribution of the model parameters *p*(θ|*D*). Monte Carlo methods, such as *Gibbs sampling,* are extremely accurate, but computationally expensive, often taking a long time to converge, and become intractable as the sample size grows. Gaussian approximation is often accurate for relatively large samples, and is more efficient than Monte Carlo methods. It is based on the fact that the posterior distribution *p*(θ|*D*) which is proportional to *p*(*D*|θ) *× p*(θ) can often be approximated as a Gaussian distribution. With more training data, the Gaussian peak becomes sharper, and tends to the MAP configuration θ_MAP_.

#### Parameter learning numerical example.

**Figure 7:**
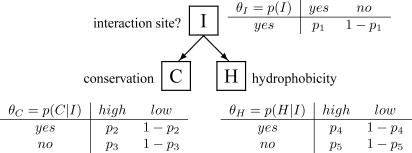
Naïve Bayes Classifier with Model Parameters in the Form of CPTs

In this numerical example, we illustrate the approaches described in the text for learning Bayesian network parameters, using the simple example of a naïve Bayes classifier to predict protein interaction sites (I) using information on conservation (C) and hydrophobicity (H). Each variable has two possible values: I = yes/no; H = high/low and C = high/low. The conditional probability tables defining the network are shown in Figure 7, and the learning problem is to determine values for the associated probabilities *p*_1–5_.

To illustrate the different methods, we will focus on parameter *p*_2_, the probability that conservation is high (C = high), given that this is a protein interaction site (I = yes). The value of *p*_2_ is to be estimated from count data; in this case, we assume that for *N* interaction sites, *n* have high conservation and *N − n* have low conservation.

**Figure 8:**
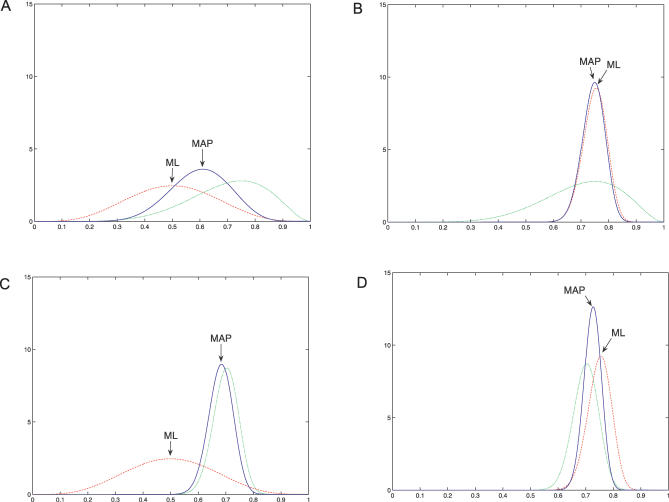
The Effects of Different Strength Priors and Training Set Sizes (A) In this case, the observed data is ten interaction sites, of which five have high conservation, five low. As expected, in this case the likelihood peaks at *p*_2_ = 0.5. The prior is *B*(7,3), indicating prior knowledge that high conservation is found in interaction sites; it corresponds to adding seven pseudocounts to the C = high category, and three to C = low, and produces a prior peaked above *p*_2_ = 0.5. The posterior is also shown, along with the MAP estimate of *p*_2_. The influence of the prior information in this case where the observed counts are low is clear. (B) Learning from 100 training examples (75 high, 25 low). Here the weak *B*(7,3) prior has little influence over the posterior distribution, and with a large training set the ML and MAP estimates are similar (*p*_2_ ∼ 0.75). The posterior distribution for *p*_2_ is narrower—some of the uncertainty about its value has been removed given the evidence (training examples). (C) Using a stronger prior *B*(70,30) still indicates that the most likely value for *p*_2_ is 0.7; however, note that the prior is narrower—a lot of evidence would be needed to be convinced that *p*_2_ was less than 0.6, say. Small samples are more susceptible to noise than larger samples. For a training set with five high and five low conservation scores, the ML estimate (*p*_2_ = 0.5) is quite different from the MAP estimate of about 0.7, which takes into account the prior. Hopefully, this illustrates why priors are useful, but also cautions against choosing the wrong prior (or too strong/weak a prior)! (D) This final example has a *B*(70,30) prior and shows ML and MAP estimates from training data with 75 high and 25 low conservation scores. This combination of a good prior and a larger training set is the example here with the least uncertainty about the value of *p*_2_.

Figure 8 describes a number of possible scenarios. In the Figure 8A–8D graphs, the red dashed line shows the likelihood, *p(data|model)*. In this case, it is derived from the binomial distribution, and represents the probability of observing *n* high conservation sites in *N* trials, as a function of the binomial parameter *p*_2_. The other graph curves are the prior *p*(*model*) (dotted green curve), giving a prior distribution for the value of *p*_2_, and the posterior *p*(*model|data*) (solid blue curve). Here we have used the beta distribution as the prior. This is a very flexible distribution on the interval [0,1]; it has two parameters *B*(*n,m*), with *B*(1,1) representing the uniform distribution and other shapes being obtained with larger and different values of *n* and *m*. An advantage of the beta distribution in this case is that when used as a prior with the binomial it yields a posterior that is also a beta distribution (but with different parameters). The beta distribution is the conjugate prior of the binomial. In fact, the *n* and *m* parameters of the beta distribution can be viewed as pseudocounts, which are added to the observed counts to reflect prior knowledge.

The Bayesian approach of calculating marginal likelihood does not involve making a point estimate of the parameter; instead, the posterior distribution is averaged over in order to fully take into account the uncertainty in the data.

## Structure Learning

Particularly in the domain of biology, the inference of network structures is the most interesting aspect; for example, the elucidation of regulatory and signalling networks from data. This involves identifying real dependencies between measured variables; distinguishing them from simple correlations. The learning of model structures, and particularly causal models, is difficult, and often requires careful experimental design, but can lead to the learning of unknown relationships and excellent predictive models.

### 

#### Full Bayesian posterior.

So far, only the learning of parameters of a BN of known structure has been considered. Sometimes the structure of the network may be unknown and this may also be learned from data. The equation describing the marginal likelihood over structure hypotheses *S^h^* as well as model parameters is an extension of Equation 7; the predictive distribution is:

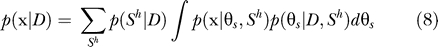



However, the computation of a full posterior distribution over the parameter space and the model structure space is intractable for all practical applications (those with more than a handful of variables).

#### Sampling methods.

Even for a relatively small number of variables, there are an enormous number of possible network structures, and the computation of a full posterior probability distribution is difficult. There are several approaches to this problem, including Markov chain Monte Carlo (MCMC) methods (such as the Metropolis–Hastings algorithm), which are used to obtain a set of “good” sample networks from the posterior distribution *p*(*S^h^*,θ*_S_|D*), where *S^h^* is a possible model structure. This is particularly useful in the bioinformatics domain, where data *D* may be sparse and the posterior distribution *p*(*S^h^,*θ*_S_|D*) diffuse, and therefore much better represented as averaged over a set of model structures than through choosing a single model structure.

#### Variational methods.

A faster alternative to MCMC is to use *variational methods* for certain classes of model. By approximating parameters' posterior distributions (which are difficult to sample from) by simpler ones, a lower bound on the marginal likelihood can be found which can then be used for model selection.

#### Structure learning algorithms.

The two key components of a structure learning algorithm are *searching* for “good” structures and *scoring* these structures. Since the number of model structures is large (super-exponential), a search method is needed to decide which structures to score. Even with few nodes, there are too many possible networks to exhaustively score each one. Efficient structure learning algorithm design is an active research area. A *greedy search* may be done by starting with an initial network (possibly with no (or full) connectivity) and iteratively adding, deleting, or reversing an edge, measuring the accuracy of the resulting network at each stage, until a local maxima is found. Alternatively, a method such as simulated annealing should guide the search to the global maximum.

There are two common approaches used to decide on a “good” structure. The first is to test whether the conditional independence assertions implied by the structure of the network are satisfied by the data. The second approach is to assess the degree to which the resulting structure explains the data (as described for learning the parameters of the network). This is done using a *score function*. Ideally, the full posterior distribution of the parameters for the model structure is computed (*marginal likelihood*); however, approximations such as the Laplace approximation or the *Bayesian Information Criterion* (BIC) score functions are often used, as they are more efficient (though approximate, and therefore less accurate). The BIC score approximates ln *p*(*D|S^h^*) as 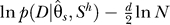 , where 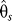 is an estimate of the model parameters for the structure, *d* is the number of model parameters, and *N* is the size of the dataset. For large *N*, the learned model often has parameters like θ_ML_. The BIC score has a measure of how well the model fits the data, and a penalty term to penalise model complexity. This is an example of *Occam's Razor* in action; preferring the simplest of equally good models. ML is not used as a score function here, as without a penalty function it would produce a completely connected network, implying no simplification of the factors.

In the case of gene regulatory networks, these structure learning algorithms may be used to identify the most probable structure to give an influence diagram for a gene regulatory network learned from data. Imoto et al. [10] derive gene networks based on BNs from microarray gene expression data, and use biological knowledge such as protein–protein interaction data, binding site information, and existing literature to effectively limit the number of structures considered to be the most biologically relevant. The fitness of each model to the microarray data is first measured using marginal likelihood, then biological knowledge is input in the form of a prior probability for structures. The posterior probability for the proposed gene network is then simply the product of the marginal likelihood of the parameters and the prior probability of the structure.

#### Causality.

Often the really interesting problems involve the learning of causal relationships [11], such as protein signalling networks [2] and gene regulatory interactions. In order to discover the underlying causal model, more than just structure learning is needed, because the available data may be insufficient to distinguish different network structures that imply the same conditional independences (Markov equivalence) and have the same score. One way to determine the directionality of the causal relations is to use intervention data, where the value of one variable is held fixed. Consider two correlated variables, X and Y, subjected to interventions (these may be expression levels of two genes, and interventions are gene knockouts). If inhibiting X leads to a limited range of observed values of Y, whereas inhibiting Y leads to a full range of X values, then it can be determined that X influences Y, but Y doesn't influence X. This implies there is a causal relationship X → Y.

Sachs et al. [2] model a protein signalling network from flow cytometry data. Simultaneous observations of multiple signalling molecules in many thousands of cells in the presence of stimulatory cues and inhibitory interventions (perturbations) and careful experimental design allow for identifying causal networks, which are potentially useful for understanding complex drug actions and dysfunctional signalling in diseased cells.

#### Dynamic Bayesian networks.

An essential feature of many biological systems is feedback. BNs are perfectly suited to modelling time series and feedback loops. When BNs are used to model time series and feedback loops, the variables are indexed by time and replicated in the BN—such networks are known as *dynamic Bayesian networks* (DBNs) [12] and include as special cases hidden Markov models (HMMs) and linear dynamical systems. The creation of experimental time series measurements is particularly important for modelling biological networks.

As an example, if in the earlier gene regulatory network example, gene G5 regulated G1, then a feedback loop (cyclic graph) would be formed. In order to perform efficient inference, BNs require a DAG to define joint probabilities in terms of the product of conditional probabilities. For probabilistic graphical models with loops, as described, either iterative methods such as loopy belief propagation must be used, or the cyclic graph must be transformed into a DAG. Assuming a (first-order) Markov process governs gene regulation, the network may be rolled out in time, to create a DBN. Generally, DBNs contain two time slices, with an instance of each variable in each time slice (*t* and *t +* Δ*t*). Directed edges are added from nodes at time *t* to the nodes they influence at *t +* Δ*t*. HMMs are a special case of DBNs, where there is a hidden set of nodes (normally discrete states), a set of observed variables, and the slices need not be time; often HMMs are used for sequence analysis and *t* is the transition from one base to the next. DBNs have been used for inferring genetic regulatory interactions from microarray data. Data from a few dozen time points during a cell cycle is a very small amount of data on which to train a DBN. Husmeier has recently used MCMC on simulated data of microarray experiments in order to access the network inference performance with different training set size, priors, and sampling strategies [13]. Variational Bayesian methods have been used to approximate the marginal likelihood for gene regulatory network model selection with hidden factors from gene expression time series data. The hidden factors capture the effects that cannot be directly measured, such as genes missing from the microarray, the levels of regulatory proteins present, and the effects of mRNA, etc. [14].

## Conclusion

Many applications in computational biology have taken advantage of BNs or, more generally, probabilistic graphical models. These include: protein modelling, systems biology; gene expression analysis, biological data integration; protein–protein interaction and functional annotation; DNA sequence analysis; and genetics and phylogeny linkage analysis. However, perhaps the most interesting application of BNs in the biological domain has been the modelling of networks and pathways. These analyses combine all the features of BNs: the ability to learn from incomplete noisy data, the ability to combine both expert knowledge and data to derive a suitable network structure, and the ability to express causal relationships. Recent application of DBNs has allowed more sophisticated relationships to be modeled; for example, systems which incorporate feedback. Furthermore, the marriage of improved experimental design with new data acquisition techniques promises to be a very powerful approach in which causal relations of complex interactions may be elucidated.

## Additional Reading

Heckerman has written an excellent mathematical tutorial on learning with BNs [9], whose notation has been adopted here. This is the suggested text to consult for statistical details and discussion of the concepts introduced in this primer. Murphy's introduction [15], along with the guide to the software Bayes Net Toolkit for Matlab, BNT [16], provides an overview of algorithms for learning. Tipping's tutorial [17] contains good illustrations of marginal likelihood, and Ghahramani's tutorial [18] contains a clear overview introducing structure learning and approximation methods. Husmeier's bioinformatics text [13] is also an excellent resource.

## Abbreviations

BN: Bayesian network
BIC: Bayesian information criterion
CPD: conditional probability distribution, CPT, conditional probability table
DAG: directed acyclic graph
DBN: dynamic Bayesian network
EM: expectation–maximisation
HMM: hidden Markov model
JPD: joint probability distribution
MAP;: maximum a posteriori
MCMC: Markov chain Monte Carlo
ML: maximum likelihood
